# NFAT5-mediated expression of S100A4 contributes to proliferation and migration of renal carcinoma cells

**DOI:** 10.3389/fphys.2014.00293

**Published:** 2014-08-08

**Authors:** Christoph Küper, Franz-Xaver Beck, Wolfgang Neuhofer

**Affiliations:** ^1^Department of Physiology, University of MunichMunich, Germany; ^2^Department of Internal Medicine, Medical Faculty of Mannheim, Medical Clinic V, University of HeidelbergMannheim, Germany

**Keywords:** NFAT5, S100A4, renal cell carcinoma, CaKi-1, cell proliferation, cell migration

## Abstract

The osmosensitive transcription factor nuclear factor of activated T-cells (NFAT) 5, also known as tonicity enhancer binding protein (TonEBP), has been associated with the development of a variety of tumor entities, among them breast cancer, colon carcinoma, and melanoma. The aim of the present study was to determine whether NFAT5 is also involved in the development of renal cell carcinoma (RCC). The most common type of RCC, the clear cell RCC, originates from the proximal convoluted tubule. We tested our hypothesis in the clear cell RCC cell line CaKi-1 and the non-cancerous proximal tubule cell line HK-2, as control. Basal expression of NFAT5 and NFAT5 activity in CaKi-1 cells was several times higher than in HK-2 cells. Osmotic stress induced an increased NFAT5 activity in both CaKi-1 and HK-2 cells, again with significantly higher activities in CaKi-1 cells. Analysis of NFAT5-regulating signaling pathways in CaKi-1 cells revealed that inhibition of the MAP kinases p38, c-Jun-terminal kinase (JNK) and extracellular regulated kinase (ERK) and of the focal adhesion kinase (FAK) partially blunted NFAT5 activity. FAK and ERK were both constitutively active, even under isotonic conditions, which may contribute to the high basal expression and activity of NFAT5 in CaKi-1 cells. In contrast, the MAP kinases p38 and JNK were inactive under isotonic conditions and became activated under osmotic stress conditions, indicating that p38 and JNK mediate upregulation of NFAT5 activity under these conditions. siRNA-mediated knockdown of NFAT5 in CaKi-1 cells reduced the expression of S100A4, a member of the S100 family of proteins, which promotes metastasis. Knockdown of NFAT5 was accompanied by a significant decrease in proliferation and migration activity. Taken together, our results indicate that NFAT5 induces S100A4 expression in CaKi-1 cells, thereby playing an important role in RCC proliferation and migration.

## Introduction

Renal cell carcinoma (RCC) was the sixth and eighth most common malignancy among men and women, respectively, in 2012, contributing to almost 14,000 deaths in the US (Siegel et al., 2012). The most common type of RCC, the clear cell RCC originates from the proximal convoluted tubule. Metastatic RCC in particular has a very poor prognosis due to the high resistance of the tumors to conventional radiation-, immune-, and chemotherapies. To develop new therapeutic strategies, significant efforts have been made in the last two decades to identify the genetic basis of clear cell RCC. In most cases of sporadic RCC, deletion or mutation of the tumor suppressor gene Von-Hippel-Lindau (VHL) can be detected (Linehan et al., 2003). Under normoxic conditions, VHL catalyzes ubiquitination and, hence, degradation of the transcription factor hypoxia-inducible factor α (HIF-α). Loss of VHL results in accumulation of HIF-α even under normoxic conditions and downstream induction of diverse growth and angiogenic factors that contribute to malignant transformation of tubular epithelial cells (Patel et al., 2006). Based on these observations, several new pharmaceutical agents that target the involved signaling pathways have been approved in recent years (Motzer, 2011). Although these agents increase the therapeutic options in the treatment of metastatic RCC, continuous research is necessary to gain a better insight to the biological basis of carcinogenesis and metastasis of renal tubular cells and to identify potential targets for new therapeutic strategies.

The aim of the present study was to determine whether the osmosensitive transcription factor nuclear factor of activated T-cells (NFAT) 5, also known as tonicity enhancer binding protein (TonEBP), is involved in the development of RCC. The NFAT protein family consists of five members (NFAT1-5) that contain a DNA-binding domain with structural similarity to the Rel-homology-region of NF-κB (Muller and Rao, 2010). While activity of NFAT1-4 is regulated by calcineurin, NFAT5 activity is modulated under hyperosmotic conditions at various levels: increased expression, increased transcriptional activity, and increased nuclear localization. Upon activation, NFAT5 binds to tonicity enhancer (TonE) elements in the regulatory region of target genes to stimulate transcription (Cheung and Ko, 2013). NFAT5 was discovered originally in the renal medulla, where it drives the expression of osmoprotective genes such as betaine-GABA-transporter-1 (BGT-1) (Miyakawa et al., 1998), aldose reductase (AR) (Miyakawa et al., 1999), sodium-myo-inositol transporter (Smit) (Miyakawa et al., 1999), taurine transporter (TauT) (Zhang et al., 2003) or heat shock protein (HSP) 70 (Woo et al., 2002), as well as genes that are part of the urinary concentrating mechanism, such as aquaporin-2 (AQP-2) and urea transporter-A (UT-A) (Han et al., 2004). Besides this function in the kidney, NFAT5 also plays important roles in other cells and tissues, partly in a tonicity-independent manner, during embryonic development, cell differentiation, inflammatory processes, and cellular stress response (Halterman et al., 2012).

Various reports have also suggested involvement of NFAT5 in the pathogenesis of various tumor entities, such as non-small cell lung cancer (Zhong et al., 2004; Mijatovic et al., 2006), melanoma (Levy et al., 2010), leiomyoma (McCarthy-Keith et al., 2011), breast cancer (Jauliac et al., 2002; Chen et al., 2009; Germann et al., 2012), or colon carcinoma (Chen et al., 2011; Slattery et al., 2011; Alvarez-Diaz et al., 2012). In colon and breast carcinoma cells NFAT5 drives the expression of the pro-metastatic factor S100A4, also known as metastasin (Chen et al., 2009, 2011). S100A4 belongs to the family of S100 proteins that consists of at least 21 calcium-binding, low-molecular-weight (10–12 kDa) proteins with no known enzymatic activity. S100 proteins form homo- or heterodimers that bind to various target proteins, thereby modulating their activities. Many studies indicated a pathophysiological role for S100A4 in the development of cancer by promoting proliferation, angiogenesis, cell motility and invasiveness (Garrett et al., 2006). S100A4 binds to several target proteins, among them the tumor suppressor p53 and the non-muscle myosin IIa. Recent studies suggest that S100A4 also plays a role in the development of RCC and may be useful as prognostic marker (Bandiera et al., 2009; Lopez-Lago et al., 2010; Wang et al., 2012; Yang et al., 2012).

In the present study, we provide evidence that NFAT5 and S100A4 are expressed abundantly in RCC cells, probably due to the constitutive activation of the extracellular regulated kinase (ERK). Under hyperosmotic conditions, NFAT5 is upregulated and, in turn, induces enhanced expression of S100A4. Knockdown of NFAT5 blunts S100A4 expression and also decreased proliferation and migration activity of the cells.

## Methods

### Materials

Pharmacological inhibitors SB202190, SP600125, U0126, SrcI-1, and PF-228 were obtained from Sigma (Deisenhofen, Germany). Anti-NFAT5 antibody, anti-Src antibody, anti-FAK antibody, and anti-phospho-FAK antibody were from Santa Cruz Biotechnology (Santa Cruz, CA, USA); anti-actin antibody was from Sigma; anti-p38, anti-phospho-p38, anti-ERK1/2, anti-phospho-ERK1/2, anti-JNK, anti-phospho-JNK, and horseradish peroxidase-conjugated anti-rabbit IgG were purchased from Cell Signaling (Beverly, MA, USA); anti-phospho–Src antibody was from Abgent (Suzhou, China); anti-S100A4 antibody was from Spring Bioscience (Pleasanton, CA, USA). Accell SMARTpool siRNA constructs for knockdown of NFAT5 or S100A4, and Accell non-targeting siRNA (#2) were obtained from Thermo Fisher Scientific (Epsom, UK). Unless otherwise indicated, other reagents were purchased from Biomol (Hamburg, Germany), Biozol (Eching, Germany), Carl Roth (Karlsruhe, Germany), or Sigma.

### Cell culture

Immortalized human proximal tubule cells HK-2 (ATCC CRL-2190) and clear cell renal carcinoma cells CaKi-1 (ATCC HTB-46) were cultured in RPMI 1640 supplemented with 10% fetal bovine serum (FBS; Biochrom, Berlin, Germany), 100 units/ml penicillin, and 100 μg/ml streptomycin (Invitrogen, Karlsruhe, Germany). Cells were grown at 37°C in a humidified atmosphere (95% air/5% CO_2_). For experiments involving pharmacological inhibitors, cells were preincubated for 30 min with the appropriate inhibitor; medium osmolality was increased by addition of NaCl.

### qRT-PCR analysis

For determination of NFAT5, S100A4, AR, and β-Actin mRNA expression levels, the total RNA from HK-2 or CaKi-1 cells was prepared by adding TRIFAST Reagent (Peqlab, Erlangen, Germany). The primers (Metabion, Martinsried, Germany) used in this experiment are:

NFAT5_fw: 5′- AAT CGC CCA AGT CCC TCT AC -3′;

NFAT5_rev: 5′- GGT GGT AAA GGA GCT GCA AG -3′;

Actin_fw: 5′- CCA ACC GCG AGA AGA TGA -3′;

Actin_rev: 5′- CCA GAG GCG TAC AGG GAT AG -3′;

S100A4_fw: 5′-CGC TTC TTC TTT CTT GGT TTG-3′;

S100A4_rev: 5′-GAG TAC TTG TGG AAG GTG GAC A-3′;

AR_fw: 5′ATC CGA GCC AAG CAC AAT AA -3′;

AR_rev: 5′-AGC AAT GCG TTC TGG TGT CA -3′

Experiments were caried out on a Roche LightCycler 480 using the SensiMix SYBR One-Step Kit (Bioline, Luckenwalde, Germany) according to the manufacturer's recommendations. Specificity of PCR product formation was confirmed by melting point analysis and by agarose gel electrophoresis.

### Immunoblot analysis

Aliquots (10–30 μg protein) were subjected to sodium dodecylsulphate polyacrylamide gelelectrophoresis (SDS-PAGE) and blotted onto nitrocellulose membranes (Amersham Pharmacia Biotech, Buckinghamshire, UK). Non-specific binding sites were blocked with 5% non-fat dry milk in PBS containing 0.1% Tween-20 (PBS-T) at room temperature for 1 h. Samples were incubated with primary antibodies in PBS-T containing 5% non-fat dry milk over night at 4°C. Subsequently, the blots were washed 3 times with PBS-T for 5 min each and the membranes then incubated with appropriate secondary antibody at room temperature for 1 h in PBS-T containing 5% non-fat dry milk. After washing with PBS-T 3 times for 5 min each, immunocomplexes were visualized by enhanced chemiluminescence (Pierce, Rockford, IL, USA).

### Reporter gene assays

Activation of NFAT5 in response to hyperosmolality was assessed using the secreted alkaline phosphatase system (SEAP), with a reporter construct, in which the SEAP open reading frame is under control of two TonE sites (Neuhofer et al., 2007). As transfection control, the vector pcDNA3-lacZ, expressing β-galactosidase under the control of the constitutive active CMV promoter, was co-transfected. For transfection, CaKi-1 or HK-2 cells were grown to ~80% confluency, trypsinated, washed in PBS and ~10^6^ cells were finally resuspended in 200 μl modified HBS electroporation buffer (0.5% HEPES, 1% glucose, 0.5% Ficoll, 5 mM NaCl, 135 mM KCl, 2 mM MgCl_2_, pH 7.4) together with 10 μg reporter vector. Electroporation was carried out with a Gene Pulser Xcell Electroporation System (Biorad, Hercules, CA, USA) at 140 V and 1000 μF (exponential decay pulse) in a 2-mm cuvette and the cells seeded immediately thereafter in 96-well plates. After growing to confluency, the cells were treated as indicated and SEAP activity in the medium determined as described in detail elsewhere (Neuhofer et al., 2007). SEAP activity was normalized to β-galactosidase activity to adjust for uneven transfection of cells.

### Knockdown of NFAT5 and S100A4

CaKi-1 cells were grown to ~80% confluency, trypsinated, washed in PBS and finally resuspended in 100 μl modified HBS electroporation buffer (see above) containing 2 μM of NFAT5 siRNA, S100A4 siRNA or unspecific Accell non-targeting siRNA (#2), as control. Electroporation was carried out as described above. Cells were incubated for 5 days, and knockdown efficiency was determined by qRT-PCR or by Western blot analysis.

### Proliferation and survival assay

For proliferation assays, CaKi-1 cells were transfected with NFAT5-specific, S100A4-specific, or unspecific (control)-siRNA constructs by electroporation as described above. After the electric pulse, cells were seeded immediately in 96-well plates at a density of ~10^4^ cells/well. After 4 days, viable cells were determined using 3-(4,5-dimethylthiazol-2-yl)-2,5-diphenyltetrazolium bromide (MTT) assay. For this purpose, cells were incubated with MTT (final concentration 0.5 mg/ml in growth medium) for 2 h at 37°C. Thereafter, growth medium was removed and formazan crystals were solubilized in 200 μl acidified isopropanol and absorption was rmeasured at 570 nm. For cell survival assays, cells were treated as described above, but after 4 days the medium osmolality was raised to 600 mosmol/kg H_2_O by addition of NaCl, and cells were incubated for an additional 24 h. Thereafter, viable cells were determined by MTT assay as described.

### Scratch assay

Cell migration was analyzed using the *in vitro* scratch assay (Liang et al., 2007), also known as the wound healing assay. CaKi-1 cells at ~80% confluency were treated for 5 days with Accell SMARTpool NFAT5 siRNA, Accell SMARTpool S100A4 siRNA, or unspecific Accell non-targeting siRNA (#2), as control, with a final concentration of 1000 nM in Accell delivery medium, containing 2% FCS, in accordance with the manufacturer's recommendations. During the 5-days incubation period, cells reached confluency. Subsequently, Accell delivery medium was replaced by RPMI 1640 medium + 10% FCS and the cell momolayer was “scratched” with a 10 μl pipette tip. Cell migration to close the scratch was monitored by capturing images in regular intervals.

### Statistical analyses

Data are expressed as means ± s.e.m. The significance of differences between the means was assessed by Student's t-test. P < 0.05 was regarded as significant. All experiments were performed at least 3 times and representative results are shown.

## Results

### NFAT5 and NFAT5 target genes are expressed abundantly in renal carcinoma cells

**Figure 1:**
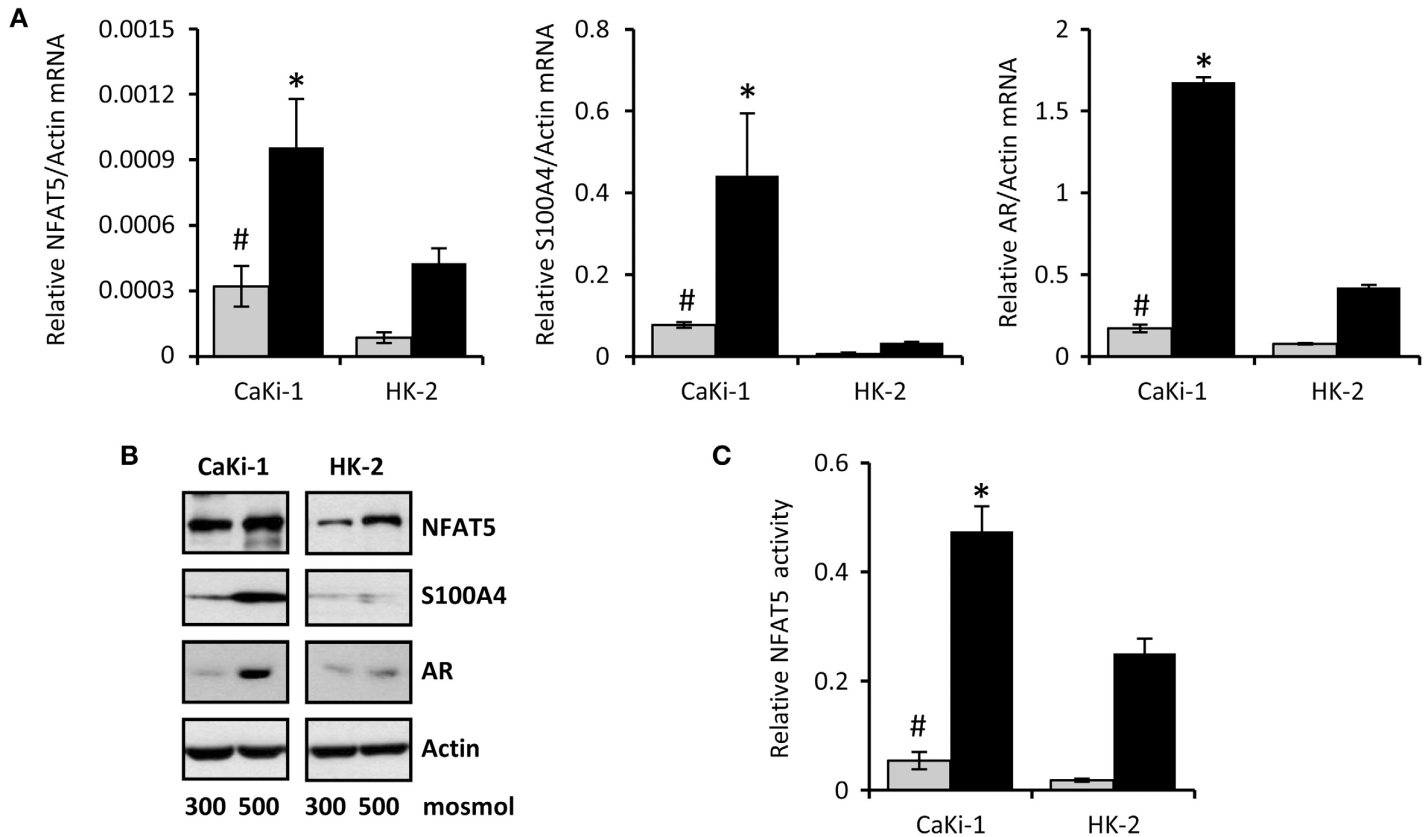
**Expression of NFAT5 and NFAT5 target genes in CaKi-1 and HK-2 cells**. CaKi-1 cells, as model for clear cell renal cell carcinoma, or HK-2 cells, as proximal tubular control cells, were kept in isoosmotic medium (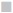; 300 mosm/kg H_2_O) or were exposed to hyperosmotic medium (■; 500 mosm/kg H_2_O). Medium osmolality was elevated by addition of NaCl. **(A)** Cells were incubated for 6 h (for determination of NFAT5 transcription) or 16 h (for determination of S100A4 and AR transcription). Thereafter, RNA was extracted and the abundance of NFAT5, S100A4, AR, and β-actin mRNA transcripts determined by qRT-PCR as described in Methods. Relative mRNA abundance of NFAT5, S100A4, or AR was normalized to that of β-actin to correct for differences in RNA input. Data are means ± s.e.m. for *n* = 4 per point; ^#^*P* < 0.05 vs. HK-2 isoosmotic medium; ^*^P < 0.05 vs. HK-2 hyperosmotic medium. **(B)** Cells were incubated for 24 h and subsequently processed for immunoblotting to determine expression of NFAT5, S100A4, and AR as described in Methods. To demonstrate comparable protein loading, the blots were also probed for β-actin. Representative blots from 4 independent experiments are shown. **(C)** Cells were transfected transiently with a reporter construct in which the SEAP gene is under control of two TonE sites. After 24 h incubation in iso- or hyperosmotic medium, SEAP activity was measured as described in Methods. Data are means ± s.e.m. for *n* = 4 per point; ^#^*P* < 0.05 vs. HK-2 isoosmotic medium; ^*^P < 0.05 vs. HK-2 hyperosmotic medium.

The cell line CaKi-1 was used as model for metastatic clear cell RCC. As non-cancerous control cells, the proximal tubule cell line HK-2 was used. Expression of NFAT5 in CaKi-1 and HK-2 cells was determined at both the mRNA (Figure 1A) and protein (Figure 1B) levels. NFAT5 levels were significantly higher in CaKi-1 cells compared to HK-2 cells. In both cell lines, NFAT5 expression increased during hyperosmotic stress (Figures 1A,B), whilst again expression levels were significantly higher in CaKi-1 cells. Expression of S100A4 was almost completely absent in HK-2 cells, while substantial amounts were present in CaKi-1 cells. Under hyperosmotic stress conditions, S100A4 expression in CaKi-1 cells increased further. As control, we also evaluated expression levels of the well-defined NFAT5 target gene AR. In accordance with the results above, expression levels of AR were also enhanced in CaKi-1 cells, presumably due to increased NFAT5 activity. Cellular activity of NFAT5 in CaKi-1 and HK-2 cells was assayed using a TonE-driven reporter vector. Cells, transiently transfected with the reporter construct, were incubated for 24 h in iso- or hyperosmotic medium. Basal NFAT5 activities under isoosmotic conditions were significantly higher in CaKi-1 cells (Figure 1C). Under hyperosmotic conditions, NFAT5 activity increased approximately 10 times in both cell types, and hence was significantly higher in CaKi-1 cells, which is in accordance with the higher expression levels of the NFAT5 target genes S100A4 and AR in these cells.

### Knockdown of NFAT5 blunts S100A4 expression in CaKi-1 cells

**Figure 2:**
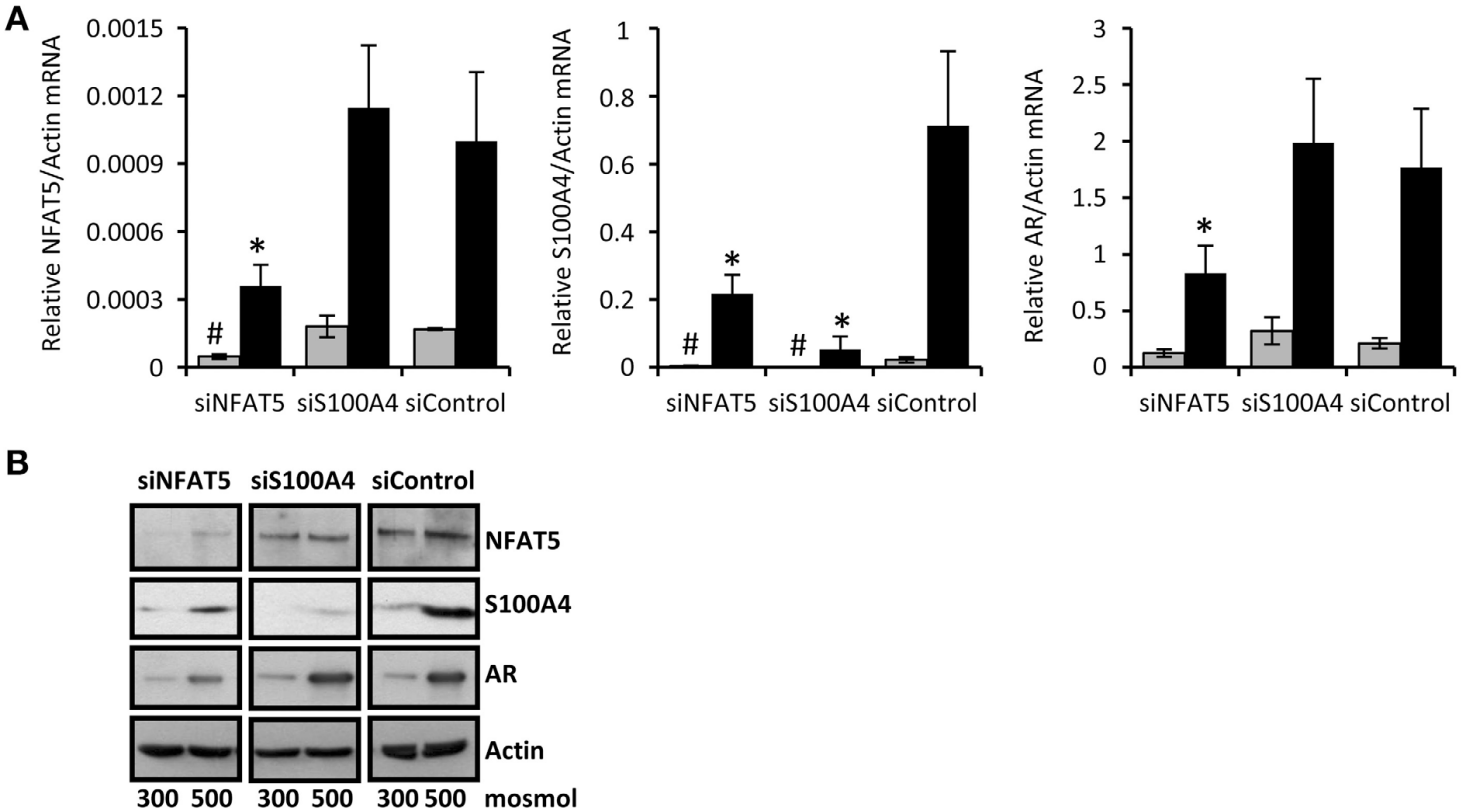
**NFAT5-knockdown attenuates S100A4 expression in CaKi-1 cells**. CaKi-1 cells were transfected with siRNA constructs for NFAT5 (siNFAT5), S100A4 (siS100A4) or with non-targeting siRNA (siControl) as indicated. Cells were kept in isoosmotic medium (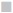; 300 mosm/kg H_2_O) or were exposed to hyperosmotic medium (■; 500 mosm/kg H_2_O). Medium osmolality was elevated by addition of NaCl. **(A)** Cells were incubated for 6 h (for NFAT5 determination) or 16 h (for S100A4 and AR determination). Thereafter, RNA was extracted and the abundance of NFAT5, S100A4, AR, and β-actin mRNA transcripts determined by qRT-PCR as described in Methods. Relative mRNA abundance of NFAT5, S100A4, or AR was normalized to that of β-actin to correct for differences in RNA input. Data are means ± s.e.m. for *n* = 4 per point; ^#^*P* < 0.05 vs. siControl isoosmotic medium; ^*^P < 0.05 vs. siControl hyperosmotic medium. **(B)** Cells were incubated for 24 h and subsequently processed for immunoblotting to determine expression of NFAT5, S100A4, and AR as described in Methods. To demonstrate comparable protein loading, the blots were also probed for β-actin. Representative blot from 4 independent experiments is shown.

To confirm that NFAT5 regulates the expression of S100A4 in CaKi-1 cells, we knocked down NFAT5 in CaKi-1 cells. Transfection of the cells with a NFAT5-specific siRNA construct resulted in an approximately 80% reduction of NFAT5 expression compared with cells transfected with an unspecific control siRNA (Figure 2). NFAT5 knockdown was accompanied by significantly reduced S100A4 expression, both under isosmotic and hyperosmotic conditions (Figure 2). Accordingly, the expression of the designated NFAT5 target gene AR was also significantly decreased in NFAT5-knockdown cells. We also performed siRNA-mediated knockdown of S100A4; as expected, this maneuver had no significant effect on expression of NFAT5 or AR. These results suggest strongly that S100A4 expression in CaKi-1 cells is under the control of NFAT5.

### Knockdown of NFAT5 and S100A4 decreases proliferation and migration of CaKi-1 cells

**Figure 3:**
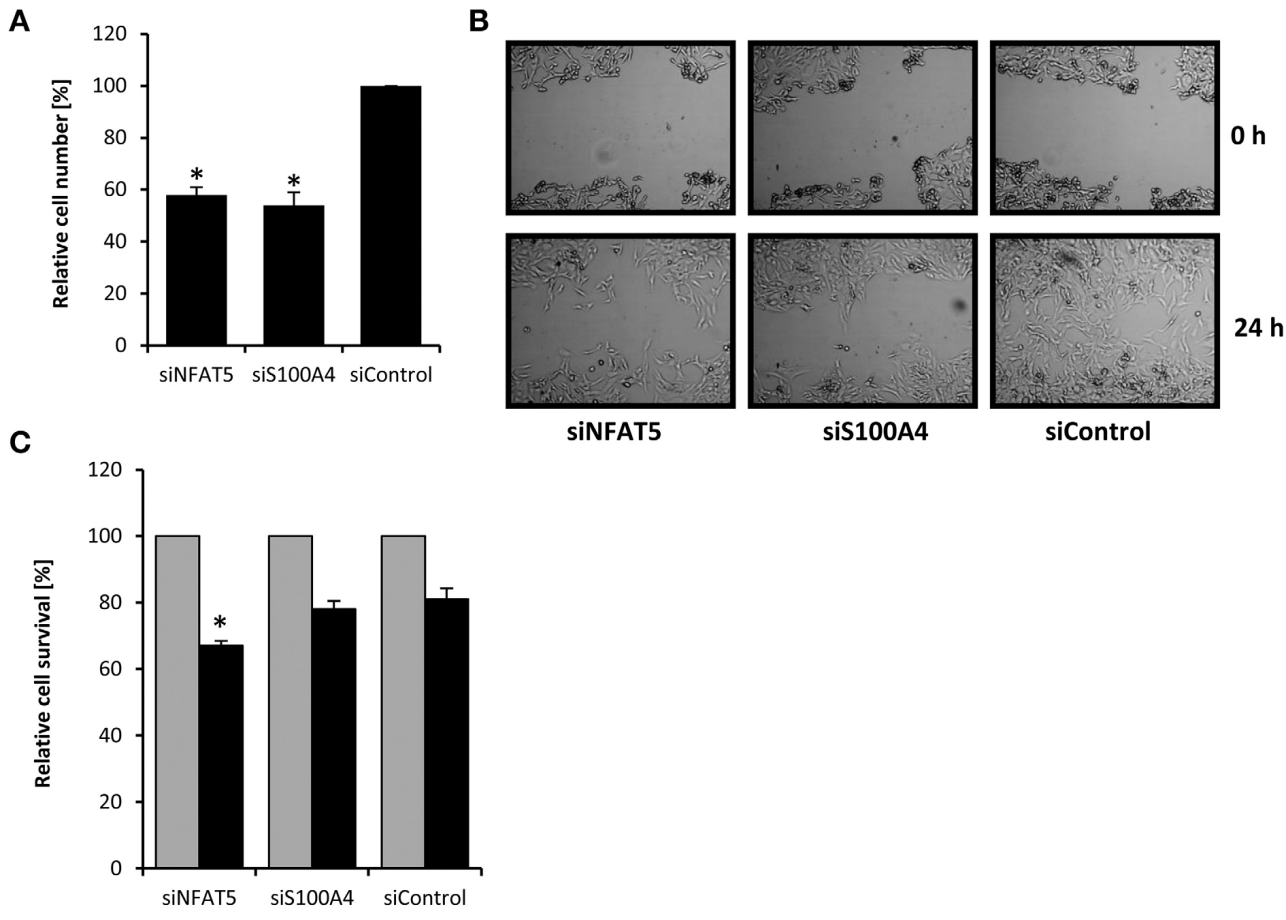
**Effect of NFAT5 and S100A4 knockdown on proliferation, migration and survival of CaKi-1 cells. (A)** Proliferation. CaKi-1 cells (10^4^ per case) were transfected with NFAT5-specific (siNFAT5), S100A4-specific (siS100A4), or unspecific control (siControl) siRNA constructs as described in Methods and seeded into one well of a 96-well plate. After 96 h, the cell number in each well was determined by MTT assay. The number of viable cells treated with control siRNA was defined as 100%. Data are means ± s.e.m. for *n* = 6; ^*^P < 0.05 vs. siControl. **(B)** Migration. CaKi-1 cells were treated with NFAT5-specific (siNFAT5), S100A4-specific (siS100A4), or unspecific control (siControl) siRNA constructs as described in Methods. After reaching confluency, the cell layer was scratched with a 10 μl pipette tip. Shown are representative phase-contrast images of cells migrating into the wounded area, immediately after scratching (0 h) and after an incubation time of 24 h. **(C)** Cell survival. CaKi-1 cells were treated with NFAT5-specific (siNFAT5), S100A4-specific (siS100A4), or unspecific control (siControl) siRNA constructs as described in Methods. Confluent cells were kept in isoosmotic medium (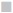; 300 mosm/kg H_2_O) or were exposed to hyperosmotic medium (■; 600 mosm/kg H_2_O) for 24 h. Thereafter, the cell number in each well was determined by MTT assay. Cell numbers in isosmotic controls (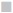) were defined as 100%. Data are means ± s.e.m. for *n* = 6; ^*^P < 0.05 vs. siControl hyperosmotic medium.

S100A4 is known to stimulate proliferation and metastasis in renal carcinoma cells (Yang et al., 2013). In the knockdown experiments described above, we noticed decelerated growth of CaKi-1 cells transfected with NFAT5- or S100A4-specific siRNA, presumably due to downregulation of S100A4. To quantify this effect, we transfected CaKi-1 cells with NFAT5-specific siRNA, S100A4-specific siRNA or unspecific control siRNA, let the cells grow for 96 h in a 96-well plate and determined the number of viable cells by MTT assay. As shown in Figure 3A, NFAT5 siRNA and S100A4 siRNA had significant inhibitory effects on CaKi-1 cell proliferation.

Next, the effect of NFAT5- and S100A4-knockdown on the migratory capacity of cells was evaluated using the *in vitro* scratch assay (Liang et al., 2007). For this purpose, a scratch was created in a confluent monolayer and cell migration to close the scratch was observed under the microscope. As can be seen in Figure 3B, knockdown of NFAT5 or S100A4 clearly decreased the migration ability of CaKi-1 cells.

Finally, the effect of NFAT5- and S100A4-knockdown on cell survival during osmotic stress was examined. Confluent cells were exposed to hyperosmotic conditions (600 mosm/kg H_2_O) for 24 h and cell survival determined by MTT assay. Compared with control cells incubated under isosmotic conditions, ~80% of CaKi-1 cells transfected with a control or S100A4-siRNA construct survived hyperosmotic stress conditions (Figure 3C), while only ~65% of NFAT5-knockdown cells survived under these conditions.

These results clearly indicate that both NFAT5 and S100A4 are important for proliferation and migration ability of CaKi-1 cells. NFAT5 is also important for survival during hyperosmotic stress, while S100A4 is probably not essential under these conditions.

### Signal transduction pathways involved in NFAT5 regulation

**Figure 4:**
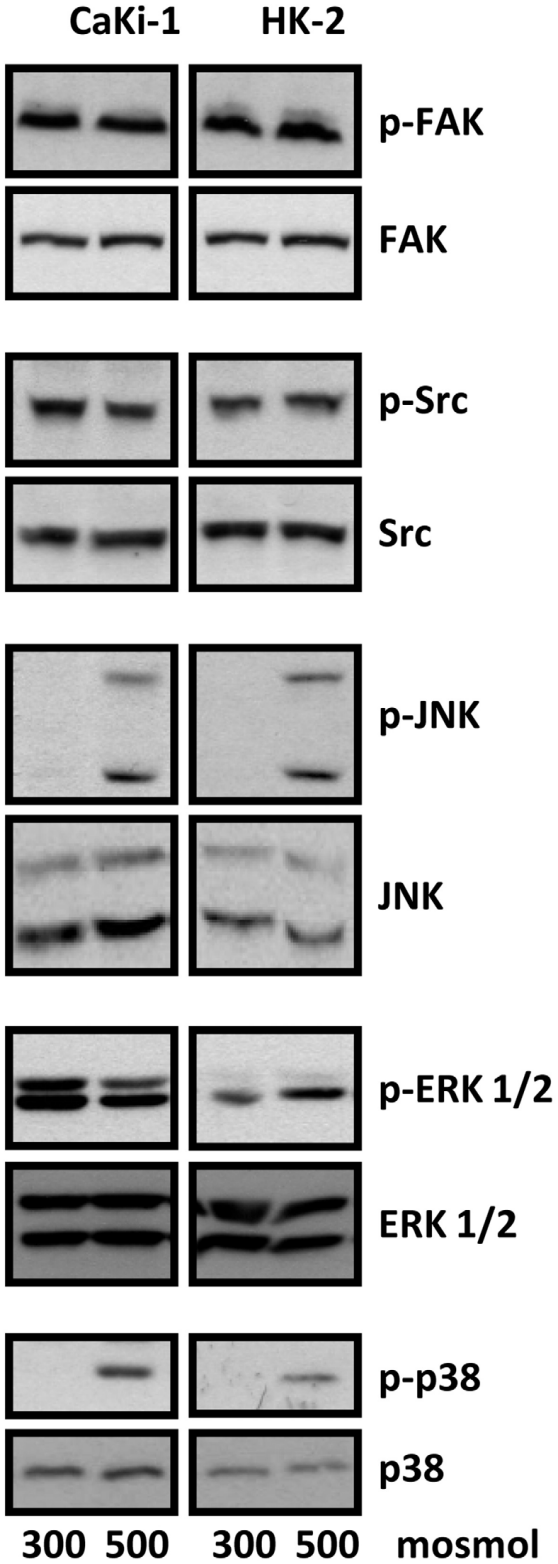
**Activation of signaling molecules in CaKi-1 and HK-2 cells**. CaKi-1 cells or HK-2 cells were incubated in isoosmotic medium (300 mosm/kg H_2_O) or hyperosmotic medium (500 mosm/kg H_2_O). Medium osmolality was elevated by addition of NaCl. Cells were incubated for 1 h and subsequently lysed and abundance and phosphorylation status of FAK, Src, JNK, ERK1/2, and p38 determined by immunoblotting as described in Methods. Representative blots from 4 independent experiments are shown.

**Figure 5:**
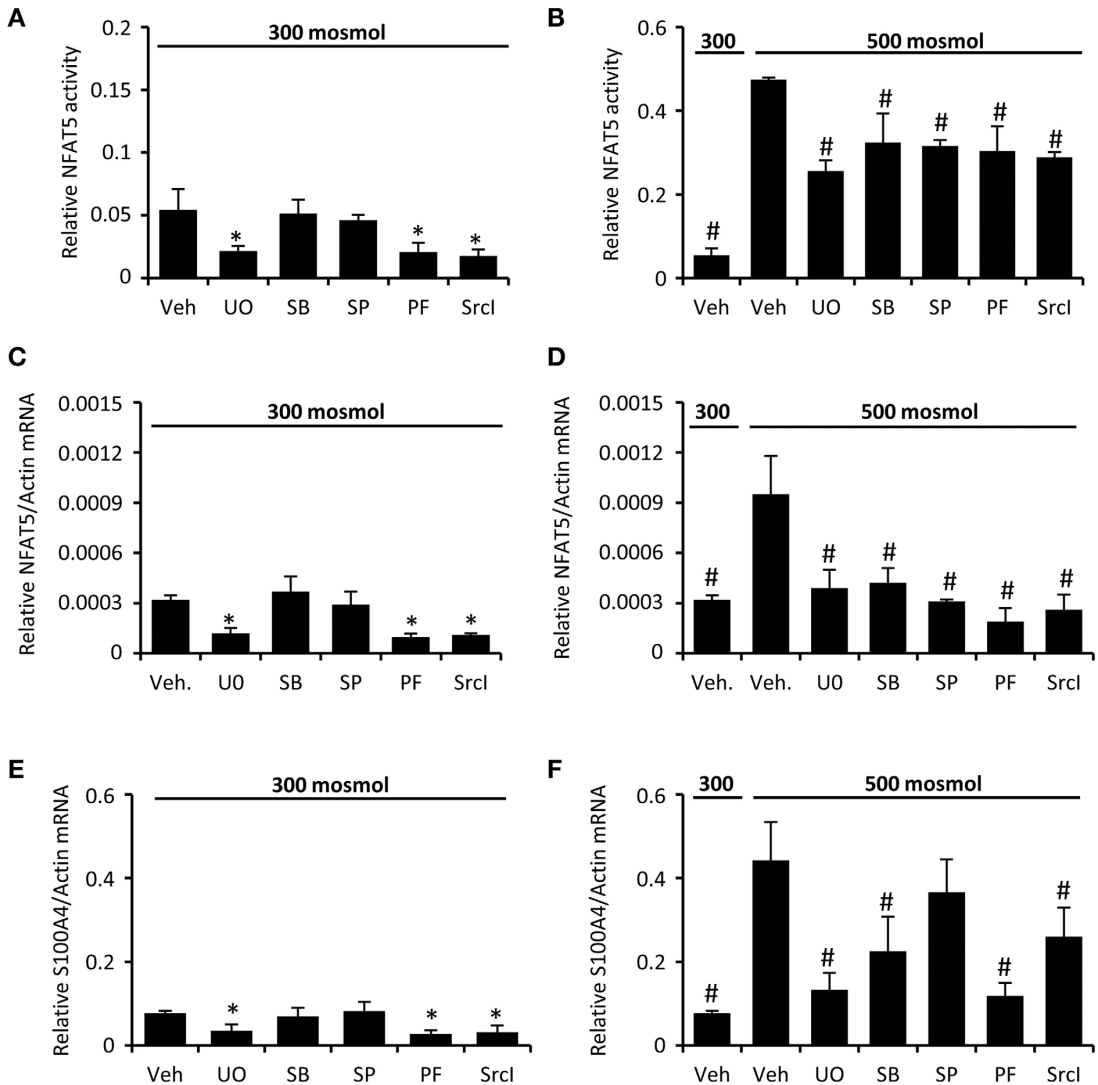
**Inhibition of FAK, Src, and MAP kinases attenuates NFAT5 activity in CaKi-1 cells**. CaKi-1 cells were preincubated with the ERK1/2 inhibitor U0126 (U0; 10 μM), the p38 inhibitor SB202190 (SB; 10 μM), the JNK inhibitor SP600125 (SP; 10 μM), the FAK inhibitor PF-228 (PF; 5 μM), the Src inhibitor SrcI-1 (SrcI; 10 μM), or vehicle DMSO (Veh) for 30 min. Subsequently, cells were incubated at 300 or 500 mosm/kg H_2_O as indicated. **(A,B)** CaKi-1 cells were transfected transiently with a reporter construct in which the SEAP gene is under control of two TonE sites. After preincubation, the transfected cells were incubated for 24 h at 300 or 500 mosm/kg H_2_O, as indicated. Subsequently, SEAP activity was measured as described in Methods. Data are means ± s.e.m. for *n* = 4 per point; ^*^P < 0.05 vs. vehicle; ^#^*P* < 0.05 vs. vehicle hyperosmotic medium. **(C–F)** After preincubation, CaKi-1 cells were incubated for 16 h at 300 or 500 mosm/kg H_2_O, as indicated. Subsequently, RNA was extracted and the abundance of NFAT5, S100A4 and β-actin mRNA transcripts was determined by qRT-PCR as described in Methods. Relative mRNA abundance of NFAT5 and S100A4 was normalized to that of β-actin to correct for differences in RNA input. Data are means ± s.e.m. for *n* = 4 per point; ^*^P < 0.05 vs. vehicle; ^#^*P* < 0.05 vs. vehicle hyperosmotic medium.

We next analyzed the activation of signal transduction pathways to elucidate the molecular basis for the high basal NFAT5 activity in CaKi-1 cells, compared to HK-2 cells. Various signaling molecules are believed to mediate NFAT5 activity, among them focal adhesion kinase (FAK) (Neuhofer et al., 2014), the SRC kinase (Chen et al., 2011), often associated with FAK, and the MAP kinases p38, ERK1/2, and JNK (Tsai et al., 2007). We found that FAK and Src kinase are constitutively active in CaKi-1 cells (Figure 4), and pharmacological inhibition of these kinases decreased NFAT5 activity and expression in CaKi-1 cells (Figure 5). However, FAK and Src kinase are also constitutively active in HK-2 cells (Figure 4), indicating that FAK and Src are necessary, but alone not sufficient to account for the high NFAT5 activity in CaKi-1 cells. The MAP kinases p38, ERK1/2 (p44/42), and c-jun-terminal kinase (JNK or p54/46) also regulate NFAT5 activity. p38 and JNK are inactive under isosmotic conditions and are activated under hyperosmotic conditions in both CaKi-1 and HK-2 cells. In contrast, ERK1/2 is constitutively active in CaKi-1 cells, even under isosmotic conditions, but not in HK-2 cells. Inhibition of ERK1/2, but not of p38 or JNK, decreased NFAT5 activity and expression under isosmotic conditions in CaKi-1 cells (Figures 5A,C), while inhibition of p38 and JNK impaired osmolality-induced upregulation of NFAT5 activity and expression (Figures 5B,D). Accordingly, osmolality induced enhancement of S100A4 was attenuated by pharmacological inhibition of Src, FAK, ERK1/2, and p38 (Figure 5F). Surprisingly, JNK inhibition attenuated hyperosmolality-induced S100A4 expression only slightly, the reason for this is unclear. Taken together, these results indicate that constitutive activation of FAK and Src is a prerequisite for NFAT5 expression, osmolality-induced activation of p38 and possibly JNK stimulate NFAT5 activation under hyperosmotic conditions, and constitutive activation of ERK1/2 in CaKi-1 cells is probably responsible for the higher basal activity and expression of NFAT5 in these cells compared with HK-2 cells.

## Discussion

The osmosensitive transcription factor NFAT5 has been associated with a variety of cancers. Here we present for the first time evidence that NFAT5 is also involved in the development and progression of clear cell RCC. We found increased expression and activity of NFAT5 in the RCC cell line CaKi-1 compared with non-cancerous proximal tubule cells. Moreover, the metastasis-associated protein S100A4, which has been previously identified in colon cancer cells as an NFAT5 target gene, is very strongly upregulated in CaKi-1 cells. Accordingly, knockdown of NFAT5 also decreased S100A4 expression. In control HK-2 cells, substantial NFAT5 expression and activity can be observed especially during hyperosmotic stress, but S100A4 expression is still almost undetectable under these conditions. This indicates that, in addition to regulation by NFAT5 activity, one or more further mechanisms control S100A4 expression. The most probable mechanism is methylation of the S100A4 promoter in normal cells, which efficiently prevents S100A4 expression, while the promoter is often hypomethylated in cancer cells, as has been observed in colon cancer cells (Chen et al., 2011). Accordingly, hypomethylation of the S100A4 promoter has also been reported in RCC cells (Lopez-Lago et al., 2010).

The role of S100A4 in RCC has not yet been studied in detail. However, in the last years three clinical studies provided evidence that high expression of S100A4 in primary tumors correlates with metastasis and poor prognosis in RCC (Bandiera et al., 2009; Wang et al., 2012; Yang et al., 2012). It has been proposed that upregulation of S100A4 mediates epithelial-to-mesenchymal transition, an initial step in the development of metastasis. Accordingly, S100A4 is highly expressed in the metastatic RCC cell line LM2, whilst knockdown decreases metastatic activity (Lopez-Lago et al., 2010). In another study on RCC cells S100A4 stimulates the expression of Bcl-2, thereby attenuating apoptosis, and MMP-2, which may also stimulate metastatic activity (Yang et al., 2013).

Interestingly, S100A4 is expressed in the normal (i.e., non-cancerous) kidney under physiological conditions (Rivard et al., 2007). While it is not expressed in the isosmotic renal cortex, S100A4 can be detected in the hyperosmotic renal medulla, where its expression is even more stimulated during antidiuresis. In the same study, the authors showed that S100A4 is strongly induced under hyperosmotic conditions in inner medullary collecting duct cells and that knockdown of S100A4 results in a 48 h-delay in the onset of adaptation to hypertonic stress. These data suggest that S100A4 is under physiological conditions part of the osmoadaptive response that allows cells to tolerate the harsh conditions in the renal inner medulla, however, in the present study knockdown of S100A4 had no significant effect on CaKi-1 cell survival during hyperosmotic stress. Whether S100A4 expression in medullary collecting duct cells is driven by NFAT5 has not been studied extensively, but the present results and other studies (Chen et al., 2009, 2011) strongly suggest that this is so. In the present study, we also observed an “intact” osmoadaptive response in CaKi-1 cells, meaning that NFAT5 mediates upregulation of osmoadaptive genes such as AR under hyperosmotic conditions. The absence of this osmoadaptive response in NFAT5-knockdown cells is probably the reason for the observed decreased resistance to hyperosmotic conditions in these cells. CaKi-1 cells, as a model for clear cell RCC, originate from the cortical proximal tubule. Since this region is not exposed to hyperosmotic stress, even during antidiuresis, osmolality-induced increase of cellular NFAT5 activity may be not relevant as long as tumor cells remain in the cortex. However, if cells of a growing tumor invade the renal medullary region, these cells are exposed to hyperosmotic conditions, which means that NFAT5 activity is increased. On the one hand, this probably facilitates survival of tumor cells by elevating the expression of S100A4 and other osmoadaptive genes like AR, HSP70, or BGT-1; on the other, the elevated S100A4 expression may also increase the metastatic activity of the tumor cells.

We observed also that NFAT5 knockdown decreases proliferation and migration of CaKi-1 cells. This is probably due, at least in part, to the decreased S100A4 expression, since it has been shown before that S100A4 promotes proliferation and migration in renal carcinoma cells (Yang et al., 2013). However, it cannot be ruled out that other NFAT5 target genes also stimulate tumor development and metastasis. Especially the designated NFAT5 target gene HSP70 has been implicated in tumor cell proliferation, differentiation, and metastasis in a wide variety of cancers (Ciocca and Calderwood, 2005). In non-small cell lung cancer, NFAT5-mediated upregulation of HSP70 confers enhanced resistance against apoptosis on tumor cells by inhibition of lysosomal membrane permeabilization (Zhong et al., 2004; Mijatovic et al., 2006). High expression of HSP70 has also been observed in RCC, however, it is not clear whether HSP70-mediated inhibition of apoptosis plays an important role in carcinogenesis and tumor progression in RCC (Ramp et al., 2007). Other proposed NFAT5 target genes reportedly upregulated in RCC are Cyr61 (O'Connor et al., 2007; Chintalapudi et al., 2008), and COX-2 (Chen et al., 2004; Favale et al., 2009), whether NFAT5 is responsible for this upregulation remains to be established.

We investigated osmolality-related signal transduction processes and observed activation of the MAP kinases p38 and JNK in both CaKi-1 and HK-2 cells in response to hyperosmotic conditions. Our results indicate clearly that p38 is involved in upregulation of NFAT5 activity and expression of the NFAT5 target gene S100A4 in CaKi-1 cells under these conditions, consistent with previous reports (Lee et al., 2008; Roth et al., 2010). The role of JNK for NFAT5 activation in CaKi-1 cells is more uncertain. Inhibiton of JNK decreased NFAT5 activity, but expression of S100A4 was only slightly and non-significantly decreased. The reason for this discrepancy is not clear. Most reports in the literature deny an involvement of JNK in NFAT5 activation (Kultz et al., 1997; Kojima et al., 2010; Roth et al., 2010).

In colon cancer cells NFAT5-mediated upregulation of S100A4 has been shown to be stimulated by Integrins and Src kinase (Chen et al., 2011). Integrins are transmembrane receptors that link the extracellular matrix to the intracellular actin cytoskeleton. Integrins can also initiate intracellular signaling events by co-clustering with receptor- and non-receptor protein tyrosine kinases (PTK). The non-receptor PTK Src often forms a dual kinase complex with the non-receptor PTK FAK. Activated FAK-Src complex promotes cell cycle progression, cell motility and cell survival and is therefore often associated with the development of cancer and metastasis (Mitra and Schlaepfer, 2006). It was reported recently that Src and FAK are also expressed and activated in patients with clear cell RCC (Qayyum et al., 2012). Our own studies suggest that FAK is a positive regulator of NFAT5 expression in HEK 293 cells (Neuhofer et al., 2014). Accordingly, the data of the present study provide evidence that NFAT5 expression and activity in CaKi-1 cells is positive regulated by Src and FAK. Pharmacological inhibition of these kinases decreased cellular NFAT5 activity and expression of S100A4, and both kinases are constitutively active in CaKi-1 cells. Since Src and FAK were also constitutively active in HK-2 cells it is doubtful whether pathophysiological upregulation of FAK-Src activity is responsible for the elevated NFAT5 activity in RCC cells. In contrast, the MAP kinase ERK1/2 was constitutively active in CaKi-1 cells, but not in HK-2 cells. The role of ERK in NFAT5 activation is currently controversial and may depend on the cell type (Tsai et al., 2007; Morancho et al., 2008). In the present study, inhibition of ERK in CaKi-1 cells attenuated both NFAT5 activation and expression and also S100A4 expression, indicating that constitutive activation of this kinase is at least partly responsible for the high NFAT5 activity in RCC cells. Constitutive activation of the Ras-Raf-MEK-ERK pathway, which mediates cellular responses to growth signals under physiological conditions, has been observed in a wide variety of cancers (Roberts and Der, 2007), but the molecular mechanisms underlying this constitutive activation vary. An important mechanism that has been identified in various tumor entities is mutation of the BRAF gene, which results in expression of a constitutive active B-Raf kinase and hence constitutive activation of ERK (Davies et al., 2002). However, since CaKi-1 cells harbor a wildtype BRAF gene (Friday et al., 2008), this mechanism can be excluded. A possible mechanism for constitutive ERK activation in RCC cells is increased expression of transforming growth factor-α (TGF-α), a ligand of the epidermal growth factor receptor (EGFR). We have previously found in (non-cancerous) renal collecting duct cells that maximal NFAT5 activation requires TGF-α-mediated activation of the EGFR and the downstream ERK kinase (Küper et al., 2009). Furthermore, overexpression of TGF-α has long been known as a characteristic feature and has been implicated with proliferation and vascularization of RCC (Gomella et al., 1989; Gunaratnam et al., 2003; Pelletier et al., 2009). We plan to investigate this potential mechanism for ERK activation in RCC cells in future studies.

## Conclusions

Taken together, the present study shows that the osmosensitive transcription factor NFAT5 is expressed abundantly and is highly active in the RCC cell line CaKi-1. NFAT5 stimulates the expression of the S100A4 metastasis factor in these cells, thereby regulating proliferation and migration activity. These results indicate that NFAT5 may contribute to the development and progression of RCC.

### Conflict of interest statement

The authors declare that the research was conducted in the absence of any commercial or financial relationships that could be construed as a potential conflict of interest.
